# Inhibition and eradication activity of truncated α-defensin analogs against multidrug resistant uropathogenic *Escherichia coli* biofilm

**DOI:** 10.1371/journal.pone.0235892

**Published:** 2020-07-14

**Authors:** Neda Moazzezy, Mohammad Reza Asadi Karam, Sima Rafati, Saeid Bouzari, Mana Oloomi

**Affiliations:** 1 Molecular Biology Department, Pasteur Institute of Iran, Tehran, Iran; 2 Immunotherapy and Leishmania Vaccine Research Department, Pasteur Institute of Iran, Tehran, Iran; Ross University School of Veterinary Medicine, SAINT KITTS AND NEVIS

## Abstract

Today the development of antibiotic resistance, especially in the treatment of bacterial infections associated with biofilms, has led to increasing the importance of antimicrobial peptides (AMPs). In this work, antimicrobial and synergistic activity of three truncated HNP-1 analogs (2Abz^14^S^29^, 2Abz^23^S^29^, and HNP1ΔC18A) with β-lactam (amoxicillin and cefixime) and fluoroquinolones (ciprofloxacin and norfloxacin) antibiotics against multidrug-resistant (MDR) uropathogenic *E*. *coli* clinical isolates were evaluated. The anti-biofilm potential of peptides at different stages was also investigated. All peptides exhibited additive activity just with β-lactam antibiotics in a checkerboard synergy assay. Inhibition and eradication of MDR uropathogenic *E*. *coli* biofilm were shown by all test peptides at different concentrations. Thus, truncated HNP-1 analogs (2Abz^14^S^29^, 2Abz^23^S^29^, and HNP1ΔC18A) may have the potential for the treatment of urinary tract infections (UTIs) caused by biofilm-forming MDR uropathogenic *E*. *coli*.

## Introduction

Today, the momentous development threat to human health is the inappropriate and excessive use of antibiotics in infectious disease therapy and the rapid emergence of resistant bacteria. For this reason, it is essential to find a proper alternative to antibiotics [1]. Antibiotics are first-line and current standard treatment of urinary tract infections (UTIs), so the presence of multidrug-resistant (MDR) bacteria is a constant problem for treating this bacterial infection. In recent years, antimicrobial peptides (AMPs) with multi-functional nature, also known as host defense peptides (HDPs), have been considered a suitable alternative to antibiotics [2]. AMPs are one of the main elements of the body's innate immunity and protect the host against pathogens in a direct or indirect manner [3]. Mammalian α-defensins are small cationic AMPs with considerable therapeutic potential that can overcome resistance problems in antimicrobial therapy [4]. Human neutrophils secrete human neutrophil peptides (HNPs) from azurophilic granules when confronted with microbial pathogens [5, 6]. The release of constitutive and inducible defensins in epithelial linings the urinary tract inhibits the adhesion of bacteria [7]. Although HNPs have proper biological activities, their considerable therapeutic limitations are the correct formation of the three disulfide pairing between cysteine residues in the chemical synthesis and the high cost of production [8]. Moreover, it is noticeable that using natural human antimicrobial peptides for treatment can increase the risk of pathogens being resistant to our innate immune peptides [9]. In the present study, we used three synthetic peptides: 2Abz^14^S^29^, 2Abz^23^S^29^ [10], and HNP-1ΔC18A [11], which exhibited a broad spectrum of antibacterial activities similar to that of the parent HNP-1. All three truncated peptides are derived from the C-terminal fragment of the HNP-1(N-ACYCRIPACI^10^AGERRYGTCIYQGRLWAFC^29^C-C).

Uropathogens can form multicellular bacterial communities coated in extracellular polymeric substances called biofilms that protect bacteria against immune responses and antimicrobial agents [12]. Therefore, the action of AMPs against bacterial biofilms may be valuable in their therapeutic potential. In the present investigation, we evaluated the antibacterial and anti-biofilm activities of three engineered peptides: 2Abz^14^S^29^, 2Abz^23^S^29^, and HNP1ΔC18A against multidrug-resistant (MDR) uropathogenic *E*. *coli*. The synergistic antibacterial effect between selected antibiotics and peptides were also investigated.

## Materials and methods

### Peptides and antibiotics

The peptides 2Abz^14^S^29^, 2Abz^23^S^29^, and HNP1ΔC18A were synthesized by biomatik (Canada) in trifluoroacetate (TFA) salt, and high-performance chromatography (HPLC) purified to > 96% with water and acetonitrile. All the peptides were dissolved in 0.01% acid acetic and were stored in the dark at -80°C until use. The main properties of peptides are listed in Table 1. The antibiotics used in this study are amoxicillin, cefixime, ciprofloxacin, and norfloxacin (Sigma-Aldrich). Stock solutions from dry powder were prepared and stored in the dark at -20°C until use.

**Table 1:** Properties of synthetic peptides.

Peptides	Amino acid sequence	Molecular formula	Molecular weight (g/mol)	Purity (HPLC)	P*I*	Charge
**2Abz^14^S^29^**	**N-**2Abz**RYGTC**(Acm)**IYQGRLWAFS-C**	**C**_**94**_**H**_**131**_**N**_**25**_**O**_**21**_**S**_**1**_	**2012.26**	**96.5%**	**9.32**	**+2**
**2Abz^23^S^29^**	**N-RYGTC**(Acm)**IYQ**2Abz**RLWAFS-C**	**C**_**94**_**H**_**131**_**N**_**25**_**O**_**21**_**S**_**1**_	**1954.20**	**98.7%**	**9.32**	**+2**
**HNP-1ΔC18A**	**N-IAAERRYATIYQARLWAF-C**	**C**_**103**_**H**_**155**_**N**_**29**_**O**_**25**_	**2199.56**	**98.4%**	**10.1**	**+2**

*PI*: Isoelectric point was calculated through the BaAMPs (biofilm-active antimicrobial peptides) database.

### Bacterial strains and media

Bacterial strains *E*. *coli* ATCC 25922, uropathogenic *E*. *coli* CFT073 and UTI89, *Pseudomonas aeruginosa* PAO1, and ATCC 27853 as references and 20 multidrug-resistant clinical *E*. *coli* isolates from UTI specimens were used. Isolates were kindly provided by the Department of Molecular biology, Institute Pasteur of Iran, Tehran, Iran. Clinical isolates were classified based on being resistant to antibiotics (amoxicillin, ciprofloxacin, cefixime, and norfloxacin). All bacterial strains were grown in Luria-Bertani (LB) broth at 37°C with aeration at 110 rpm in 15-ml tubes.

### Measurement of the Minimum Inhibitory Concentration (MIC)

Antimicrobial activity of peptides and antibiotics was performed by the broth microdilution method according to a previously reported protocol [13]. In short, the bacterial strains, freshly inoculated in Mueller-Hinton broth (MHB, Difco, Becton Dickinson Co., Sparks, MD, USA) from overnight culture to reach the exponential phase. Antimicrobial agents were serially diluted and then adjusted bacterial suspensions at a final concentration of 5 × 10^5^ CFU/ml were added to each well of 96 well U-bottom polystyrene microtiter plates (Grainer, Germany). The plates were incubated at 37°C for 18 to 24 h. The MIC value was determined as the lowest concentration of an antimicrobial agent that entirely prevented visible growth.

### Determination of Fractional Inhibitory Concentrations (FIC_s_)

For determining FIC values, the effects of test peptides in combination with each other on UPEC CFT073 and with traditional antibiotics on multi-drug resistant (MDR) uropathogenic *E*. *coli* isolates were evaluated by a checkerboard titration method [14]. The two-fold serial dilutions (1/8 × MIC to 2 × MIC) of peptides were added in the presence of two-fold serial dilutions (1/32 × MIC to 2 × MIC) of antibiotics to the microtiter plates. Antibiotics were distributed alone in the first row and were mixed with peptides in the remaining rows. Peptides were also dispensed alone in the first column. Bacteria inoculum was prepared according to the broth microdilution assay to adjust a final concentration of 5 × 10^5^ CFU/ml. After adding the bacteria to the wells, plates were incubated for 24 h at 37°C. The checkerboard assay results were analyzed in terms of the ΣFIC, which was calculated using the formula: ΣFIC = FIC (A) (MIC of drug A in the presence of drug B/MIC of drug A alone) + FIC (B) (MIC of drug B in the presence of drug A /MIC of drug B alone). Results were defined to be synergistic (ΣFIC ≤ 0.5), additive (0.5 < ΣFIC ≤ 1.0), indifferent (1.0 < ΣFIC ≤ 4.0), or antagonistic (ΣFIC > 4.0). All the experiments were repeated twice.

### Time killing assay

The time-dependent killing of the MDR uropathogenic *E*. *coli* isolate exposed to 2Abz^14^S^29^, 2Abz^23^S^29^, and HNP1ΔC18A was estimated by measuring the reduction in the numbers of CFU/ml for 3 h. The bacterial strain was grown overnight in LB, and then the bacterial concentration was adjusted to 5 × 10^5^ CFU/ml in fresh LB medium. Peptides at 1 × MIC were added to diluted bacteria in 96-well microtiter plates at 37°C. Aliquots at various time points (0, 60, 90, 120, and 180 min) were taken, subsequently diluted in sterile phosphate buffer saline (PBS) and plated on LB agar plates. Bacterial colonies formed were counted after incubation of plates for 24 h at 37°C. The bacterial growth was shown by plotting the CFU/ml against incubation time. The results were displayed as the mean data from two independent assays.

### Crystal violet assay

The evaluation of the total biomass of biofilms was performed by the crystal violet (CV) staining. The aim of this assay was detecting for potent biofilm producer strains among MDR *E*. *coli* isolates. All clinical *E*. *coli* isolates (n = 20) were incubated overnight at 37°C in 5 ml LB broth. Biofilm formation was performed by aliquoting of 100 μl of the bacterial suspension (10^7^ CFU/ml) into wells of flat-bottom polystyrene 96-well microtiter plate (Grainer, Germany) and incubated at 37°C for 18 h. After the incubation, the waste media was removed, and plates were rinsed three times with 200 μl PBS solutions, air-dried, and stained with 0.1% (w/v) CV for 20 min at room temperature. The dye was removed and then air-dried stained biofilms were solubilized with 125 μl a mixture of the ethanol and acetone (80%/20%) for 20 min. Then, the biofilm cell-associated dye was measured at 570 nm using the Epoch reader (BioTek). Biofilm assays were conducted three times in three independent experiments, and each assay was carried out in three separate wells. We found that CV absorbance of just one *E*. *coli* isolate to be ≥ 1.5, which indicates that the test bacterial isolate has a high biofilm-forming capability. In the following, we continued anti-biofilm studies by antimicrobial agents on this *E*. *coli* isolate.

### Crystal violet biofilm inhibition assay

A biofilm-inhibition assay was used to determine the capacity of the peptides (2Abz^14^S^29^, 2Abz^23^S^29^, and HNP1ΔC18A), or antibiotics (amoxicillin and cefixime) to prevent or reduce biofilm formation against MDR *E*. *coli* isolate and *P*. *aeruginosa* PAO1, which have the high biofilm-forming ability. Bacterial cells from the overnight culture were inoculated into a fresh MHB media (1.100 dilutions). Antimicrobial agents were added at time zero (before adding the diluted, overnight cultures) into wells of 96-well microtiter plate at different concentrations. The plates were then incubated at 37°C for 24 h to allow biofilm formation. After the incubation, media and planktonic cells were removed, and each well was washed twice with sterile distilled water for removal of the free-floating cells. The plates were dried, and biofilm cells were stained with 0.1% CV for 15 min at room temperature. The excess of stain was removed, and all wells were rinsed three times. Then, the attached dye was solubilized by adding a mixture of the ethanol and acetone (80%/20%) to the wells. Finally, after 20 min incubation, optical density (OD_570_ nm) values were measured using a microtiter plate reader. Percent inhibition of biofilm biomass of peptides and antibiotics alone and in combination was estimated using the following formula [15]. Experiments were repeated three times. % Inhibition of biofilm biomass = OD _control_−OD _treatment_ /OD _control_ ×100.

### Crystal violet biofilm eradication assay

The ability of the peptides (2Abz^14^S^29^, 2Abz^23^S^29^, and HNP1ΔC18A), or antibiotics (amoxicillin and cefixime) to eradicate preformed biofilm of MDR *E*. *coli* isolate and *P*. *aeruginosa* PAO1 was also investigated. Briefly, bacteria grown overnight were diluted 1.100 in MHB and incubated in 96-well microtiter plates at 37°C for 24 h. The 24 h-old biofilms in 96-well plates were washed and air-dried. Serial two-fold dilutions of antimicrobial agents in MHB were prepared. Antimicrobial agents (100 μl individual antimicrobial agent) or (50 μl peptide plus 50 μl antibiotic) were added to each well. Plates were then incubated for further 24 h at 37°C. After incubation, the crystal violet assay was used to measure biofilm reduction according to the formula above.

### Combination therapy analysis

The anti-biofilm efficacy of combination test peptides and amoxicillin or cefixime was conducted by crystal violet assay as described before. Two-fold serial dilutions of antibiotics were tested in the presence of a fixed concentration of peptides (1/2 MIC).

### Statistical analysis

Data were statistically analyzed using SPSS software: Version 18.0. Student's t-test was applied for the analysis of data with a level of significance set at *p*<0.05.

## Results

### Minimal Inhibitory Concentration determination for bacterial species

The bacterial isolates used in this study and their MIC values are shown in Table 2. MIC values of 2Abz^14^S^29^, 2Abz^23^S^29^, and HNP-1ΔC18A for *E*. *coli* (ATCC 25922), uropathogenic CFT073, and uropathogenic UTI89 were 62.5, 125, and 125μg/ml, respectively. A set of 20 multidrug-resistant (MDR) *E*. *coli* isolates from urinary tract infections were evaluated (S1 Table). All clinical isolates were resistant to amoxicillin, ciprofloxacin, cefixime, and norfloxacin antibiotics. The results showed that all MDR clinical isolates had a MIC to 2Abz^14^S^29^ between 62.5and 125μg/mL, to 2Abz^23^S^29^ and HNP-1ΔC18A between 125 and 250μg/ml. The bacterial strain *P*. *aeruginosa* 27853 and PAO1 showed MIC values of 62.5μg/mL for 2Abz^14^S^29^ and 125μg/mL for 2Abz^23^S^29^ and HNP-1ΔC18A.

**Table 2:** Minimum Inhibitory Concentration (MIC) values of test peptides (2Abz^14^S^29^, 2Abz^23^S^29^, and HNP-1ΔC18A) and antibiotics (amoxicillin, ciprofloxacin, cefixime, and norfloxacin) against standard and clinical *E*. *coli* isolates.

Antimicrobial agents	*E*. *coli* 25922	*E*. *coli CFT073*	*E*. *coli* UTI89	Clinical *E*. *coli* isolates (N = 20)	*P*. *aeruginosa* 27853	*P*. *aeruginosa* PAO1
**2Abz^14^S^29^**	62.5	62.5	62.5	62.5–125	62.5	62.5
**2Abz^23^S^29^**	125	125	125	125–250	125	125
**HNP-1ΔC18A**	125	125	125	125–250	125	125
**AMX**	3.9(S)	3.9 (S)	3.9 (S)	>256(R)	>128(R)	>512(R)
**CFM**	1(S)	0.5(S)	0.5(S)	>256(R)	>128(R)	>16(R)
**CIP**	0.003(S)	0.05(S)	1(S)	32-125(R)	0.1(S)	0.1(S)
**NOR**	0.1(S)	0.3(S)	0.5(S)	128-256(R)	16(R)	0.1 (S)

AMX: Amoxicillin; CFM: Cefixime; CIP: Ciprofloxacin; NOR: Norfloxacin. CLSI (Clinical and Laboratory Standards Institute) breakpoints for *E*. *coli*, susceptible (S) and resistant (R) to Amoxicillin was ≤8 and >32, to Cefixime ≤8 and >32mg/l, to Ciprofloxacin ≤8 and >32 and to Norfloxacin ≤4 and >16mg/l, respectively.

### Combined effect of AMPs and antibiotics

**Fig 1:**
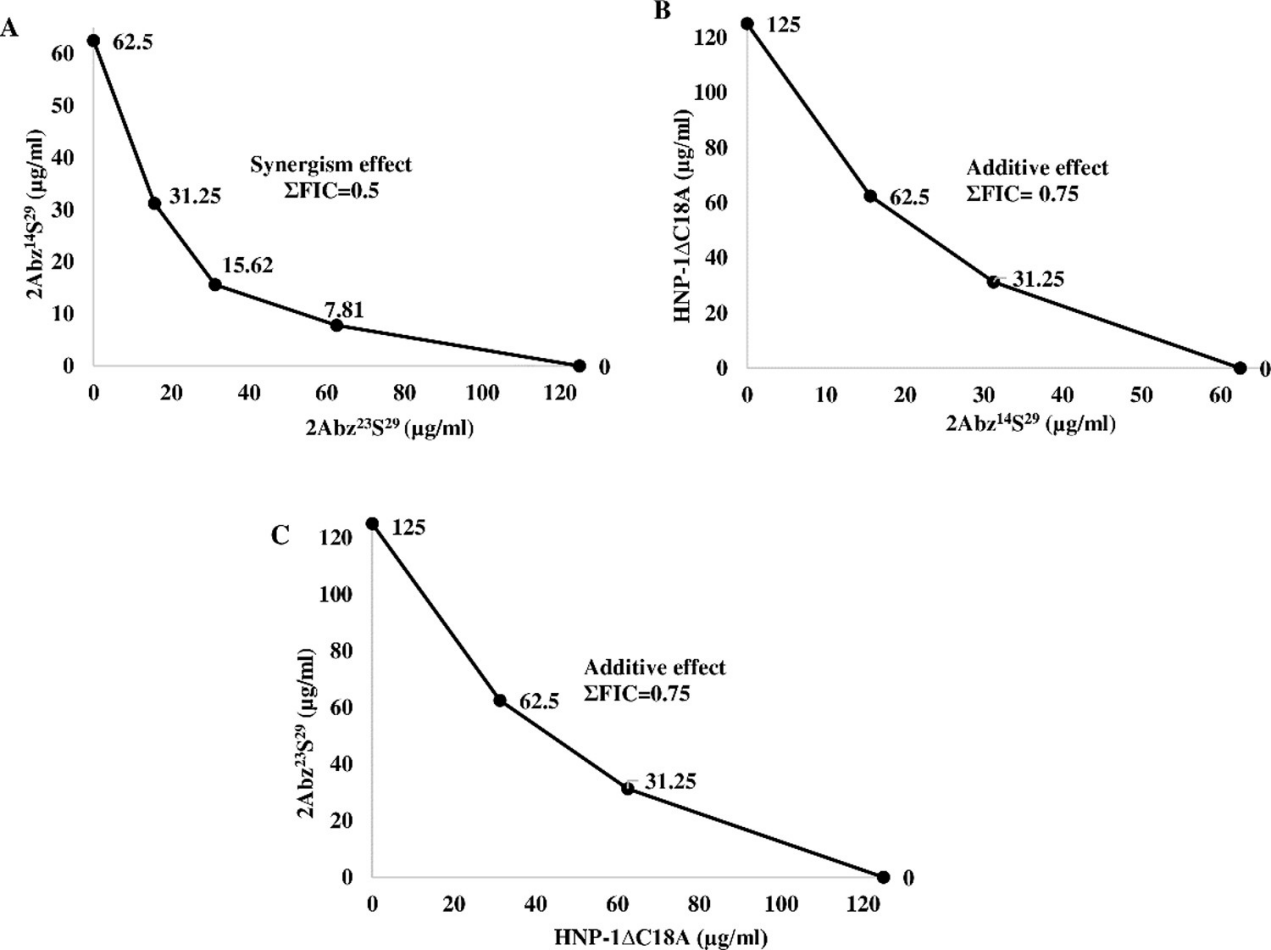
Fractional Inhibitory Concentrations (FICs) plot for 2Abz^14^S^29^, 2Abz^23^S^29^, and HNP-1ΔC18A combination against CFT073 uropathogenic *E*. *coli*. A) The ΣFIC by the 2Abz^14^S^29^ and 2Abz^23^S^29^ is (15.62/62.5) + (31.25/125) = 0.5, B) The ΣFIC by the 2Abz^14^S^29^ and HNP-1ΔC18A is (15.25/62.5) + (62.5/125) = 0.75, C) The ΣFIC by the 2Abz^23^S^29^ and HNP-1ΔC18A is (15.25/62.5) + (62.5/125) = 0.75, MIC: minimal inhibitory concentration. ΣFIC ≤ 0.5, synergistic; 1>ΣFIC> 0.5, additive; 1< ΣFIC> 4.0, indifference, ΣFIC> 4.0, antagonistic.

**Fig 2:**
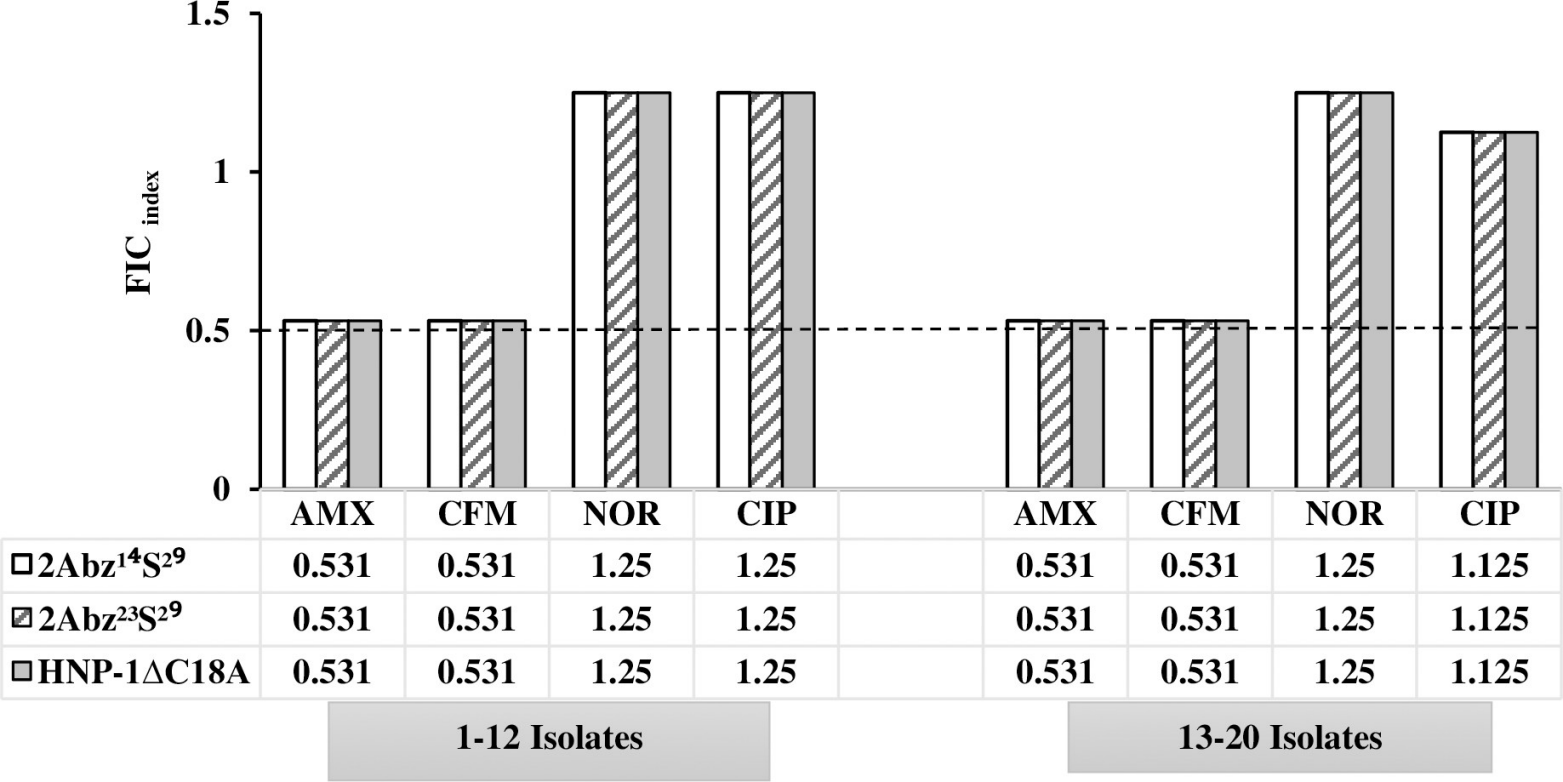
Fractional Inhibitory Concentrations (FICs) indices of combinations consisting of antibiotics and either of three antimicrobial peptides against MDR clinical *E*. *coli* isolates (n = 20). Black dashed line denotes the cutoff FIC_index_ of ≤0.5 for synergistic interaction. FIC_index_≤0.5, synergistic; 1>FIC_index_>0.5, additive; 1<FIC_index_>4.0, indifference, FIC_index_>4.0 antagonistic. See Supplementary Material (S1 and S2 Tables) for MIC and FIC values.

The results of FICs values of test peptide combinations against UPEC CFT073 are shown in Fig 1. It was observed that the combination of 2Abz^14^S^29^ and 2Abz^23^S^29^ exhibited a synergistic effect (ΣFIC = 0.5) (Fig 1A), while the combination of HNP-1ΔC18A and 2Abz^14^S^29^ or 2Abz^23^S^29^ displayed additive activity (ΣFIC = 0.75) (Fig 1B and 1C). Checkerboard assay was also performed on combinations of AMPs (2Abz^14^S^29^, 2Abz^23^S^29^, and HNP-1ΔC18A) and either β-lactam (amoxicillin and cefixime) or fluoroquinolone (ciprofloxacin and norfloxacin) antibiotics against MDR uropathogenic *E*. *coli* isolates to elucidate the interaction between them. From Fig 2, it was observed that with an FIC index of ≤0.5 as borderline, β-lactam and fluoroquinolone antibiotics did not exhibit synergistic effects with peptides against the studied bacteria, having ΣFIC ranging from 0.53 to 1.25 values. The combination of β-lactams and AMPs was additive, while the fluoroquinolones exerted indifferent effects on all AMPs.

### Killing kinetics of peptides

**Fig 3:**
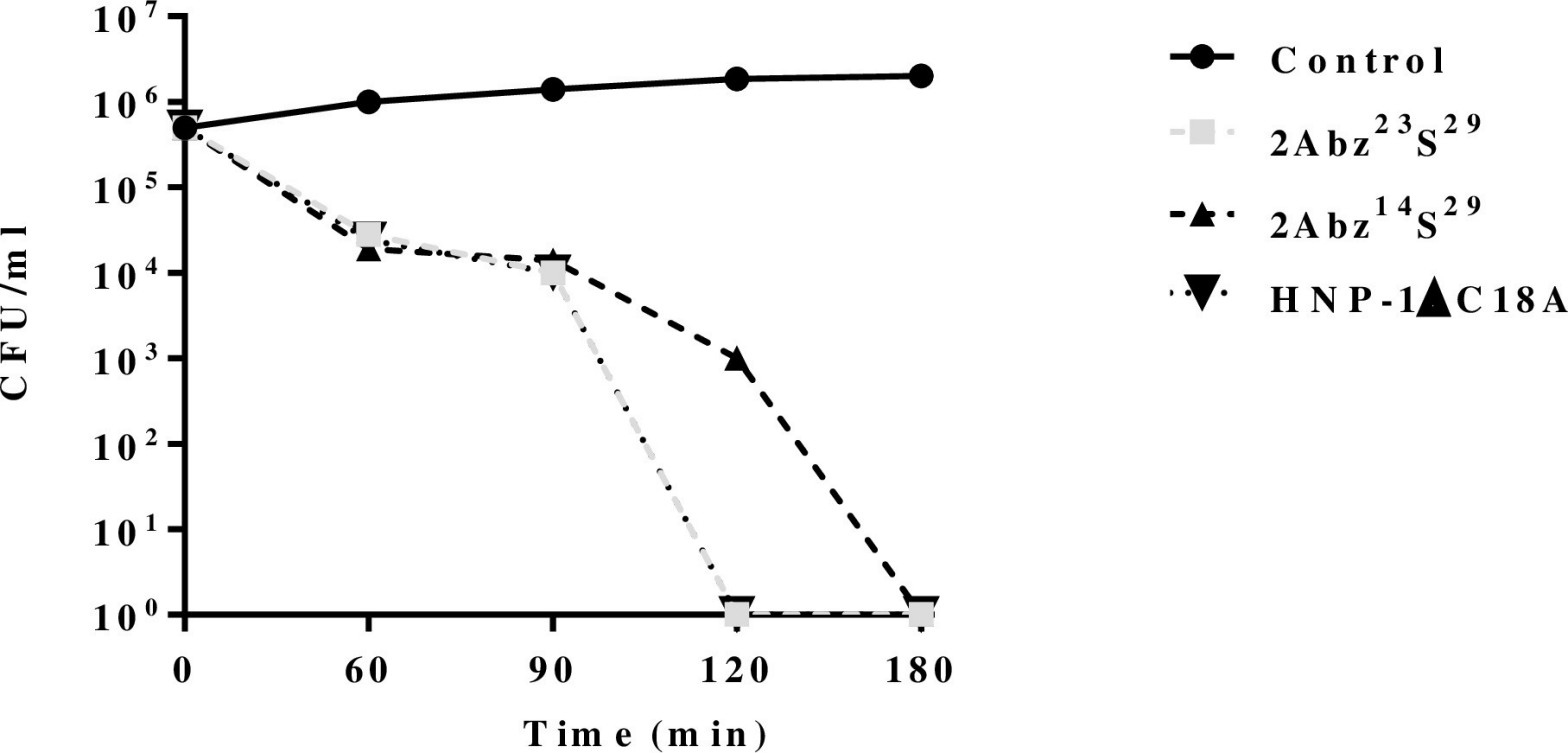
Killing kinetics of 2Abz^14^S^29^, 2Abz^23^S^29^, and HNP-1ΔC18A against multidrug-resistant *E*. *coli*. Bacteria were incubated with the peptides at concentrations equal to their MICs for different times. Control represents untreated bacteria. Data are expressed as mean of at least two independent experiments.

Time-kill studies on the MDR *E*. *coli* isolate were carried out using the AMPs, at MIC concentrations, by monitoring CFU counts for 180 min (Fig 3). Both 2Abz^23^S^29^ and HNP-1ΔC18A displayed similar killing kinetics, and also they showed faster killing kinetics than 2Abz^14^S^29^. The 2Abz^23^S^29^ and HNP-1ΔC18A peptides eliminated bacteria within 120 min, while 2Abz^14^S^29^ required 180 min to entirely killing initial inoculum. All three peptides showed a reduction in > 2 log_10_ CFU/ml decrease (99% reduction) in the number of viable bacteria at 90 min in comparison with untreated control.

### Inhibition and eradication of biofilm formation

**Fig 4:**
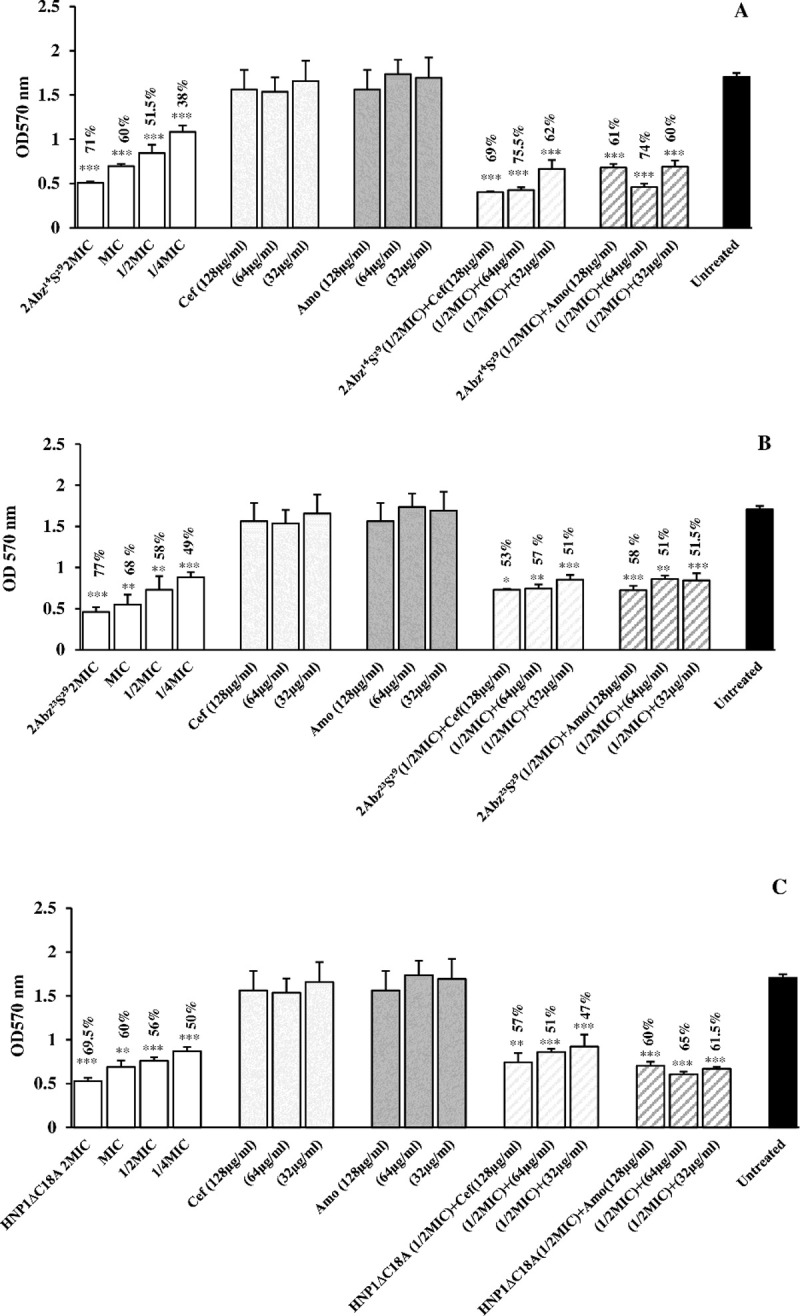
**Biofilm inhibition by individual antimicrobial peptides (2Abz**^**14**^**S**^**29**^**, 2Abz**^**23**^**S**^**29**^**, and HNP1ΔC18A), antibiotics (Cefixime and Amoxicillin) or their combination are shown in panels A, B, and C.** Untreated bar indicates *E*. *coli* biofilm without any antimicrobial agent. Data show the means ± SD of three independent experiments. An asterisk indicates the statistically significant difference between antimicrobial agent and untreated control as measured by Student’s t-test (*p < 0.05, **p < 0.01 and ***p < 0.001). Percent numbers indicate % reduction of biofilm.

The results of the anti-biofilm efficacy of test peptides and antibiotics alone and in combination at different concentrations on initial biofilm formation are shown in Fig 4. As compared with untreated controls (bacteria incubated in medium only), the different levels of peptides exhibited concentration-dependent biofilm inhibition. From Fig 4A, it was observed that the test 2Abz^14^S^29^ caused around 38% to 71% decrease of the biofilm biomass at the concentration of 31.25 up to 250 μg/ml (1/4×MIC to 2×MIC). Fig 4B and 4C showed that 2Abz^23^S^29^ and HNP-1ΔC18A at the amount of 62.5 up to 500 μg/ml (1/4×MIC to 2×MIC) reduced biomass 50–70%.

**Fig 5:**
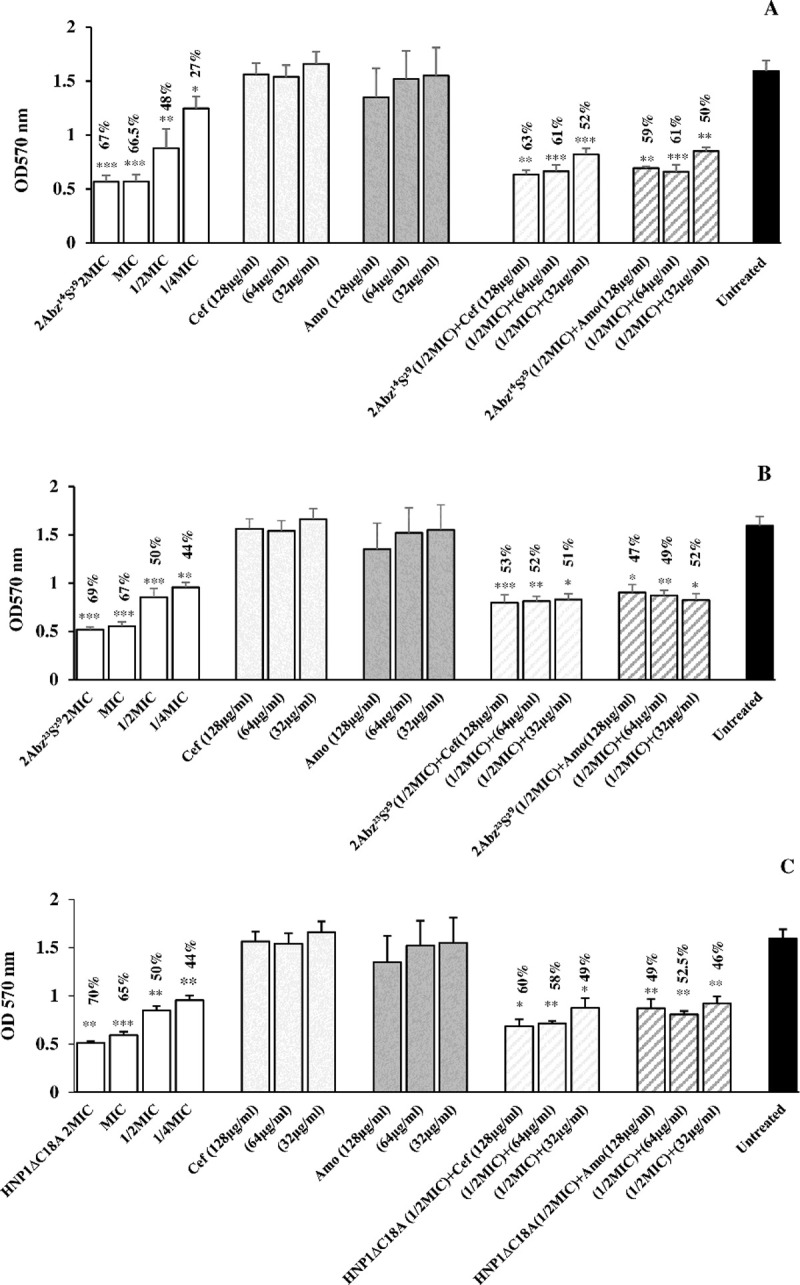
**Biofilm eradication by individual antimicrobial peptides (2Abz**^**14**^**S**^**29**^**, 2Abz**^**23**^**S**^**29**^**, and HNP1ΔC18A), antibiotics (Cefixime and Amoxicillin) or their combination are shown in panels A, B, and C.** Untreated bar indicates *E*. *coli* biofilm without any antimicrobial agent. Data show the means ± SD of three independent experiments. An asterisk indicates the statistically significant difference between antimicrobial agent and untreated control as measured by Student’s t-test (*p < 0.05, **p < 0.01 and ***p < 0.001). Percent numbers indicate % reduction of biofilm.

The eradication of mature biofilm was also studied by different concentrations of test peptides and antibiotics alone and in combination (Fig 5). From Fig 5A, quantification of biofilm biomass showed that test 2Abz^14^S^29^ caused around 27% to 67% decrease of the biofilm biomass at the concentration of 31.25 up to 250 μg/ml (1/4×MIC to 2×MIC). Fig 5B and 5C showed that 2Abz^23^S^29^ and HNP-1ΔC18A at the concentration of 62.5 up to 500 μg/ml (1/4×MIC to 2×MIC) reduced biomass 44–70%. The results of anti-biofilm efficacy of test peptides and antibiotics (amoxicillin or cefixime) combination displayed effective biofilm reduction, whereas antibiotics alone did not affect biofilm reduction (Figs 4 and 5). As shown in S1 and S2 Figs, in the case of *P*. *aeruginosa* PAO1 biofilm, 2Abz^14^S^29^ were also found 75% inhibition at 62.5 μg/ml (MIC), whereas 2Abz^23^S^29^ and HNP1ΔC18A reduced biofilm up to 84% at 500 μg/ml (4×MIC) and did not represent a significant anti-biofilm activity at lower concentrations. A similar trend was also shown for mature biofilm treated with test peptides.

## Discussion

Urinary tract infections (UTIs) are the most common bacterial diseases in humans. A highly heterogeneous group of Uropathogenic *E*. *coli* (UPEC) is the most likely causative pathogen for UTIs [16]. Uropathogens adhere to an inert or living surface, and form structures called biofilms [17]. The dispersal and resistance of biofilms, as well-developed structured sessile communities of bacterial cells, play a substantial role in UTIs [18]. Antibiotics have been prescribed for years as the only treatment agents for UTIs. However, the enhancement of antibiotic-resistant uropathogens and the prevalence of antibiotic-resistant mechanisms cause the urgent need for alternative treatment agents and strategies. Antimicrobial peptides (AMPs) and their derivatives can be appropriate candidates for antibiotic alternatives due to the possession of rapid bacterial killing kinetics, broad-spectrum antimicrobial properties, and low resistance [19]. In this study, we tested MIC values with the microdilution method as the gold standard for considering the antimicrobial activities of truncated HNP-1 analogs against some standard and clinical isolates. Kill-kinetics study was also performed for about the time course of the efficacy of test peptides against MDR uropathogenic *E*. *coli*. AMPs with rapid bactericidal rate have own benefits over conventional antibiotics, including, restricting the spread of infection, resulting in better health outcomes, minimizing emergence and likelihood of bacterial resistance and shortening the course of treatment [20]. Based on our killing kinetic results, all peptides; 2Abz^23^S^29^, HNP1ΔC18A, and 2Abz^14^S^29^ showed fast killing activity, and eliminated the initial inoculum within 120, 120, and 180 min, respectively. In the case of our study (accepted for publication), we also observed that 2Abz^14^S^29^ eliminated the initial inoculum within 90 min, and 2Abz^23^S^29^ or HNP1ΔC18A was required 120 min to kill uropathogenic CFT073 bacteria. 2Abz^14^S^29^ exhibited faster bactericidal activity against susceptible strain than resistant bacteria. Similarly, Varkey et al [11] have also shown that truncated forms of HNP-1 without cysteine, HNP1ΔC18A, have a higher bactericidal rate against *E*. *coli* and *P*. *aeruginosa* than HNP-1. However, more time-kill studies against several clinical isolates would be assessed in the future.

Combination therapy has been offered in recent years as more powerful strategies to counter infections, including MDR bacterial pathogens. The beta-lactams, including amoxicillin and cefixime antibiotics that inhibit bacterial cell wall synthesis, and fluoroquinolones, including ciprofloxacin and norfloxacin antibiotics which act by inhibiting DNA synthesis, are some of the most effective agents for microbial infections. In our study, the checkerboard results demonstrated that amoxicillin or cefixime antibiotics had an additive effect with each peptide, but none of the peptides had a synergistic effect with ciprofloxacin or norfloxacin antibiotics against MDR *E*. *coli* isolates. One of the possible reasons for the lack of synergistic combination could be that these peptides are unable to penetrate the bacterial inner membrane, which is the essential site of the effects of the peptides on the bacterial cells [11]. Contrary to our results, Wang et al [21] have shown that HNP-3 and ciprofloxacin combination had a strong synergistic effect against *P*. *aeruginosa*-resistant strain and caused by the enhancement in the efflux of intracellular ATP and permeability by HNP-3. Another recent report showed that there was not any synergistic antibacterial effect between HNP-1 and rifampicin, gentamicin, or ofloxacin, which affect intracellular biosynthesis of proteins and nucleic acids. Using HNP-1 at concentrations equal to1/4×MIC had no significant membrane damaging effect against *E*. *coli* ML-35p [22]. The combined interaction of AMPs and antibiotics may be strain-dependent [23]. In light of this, it is necessary to continue further studies on various bacterial types to obtain precise and reliable conclusions.

In the present investigation, the anti-biofilm activity of individual truncated HNP-1 analogs and conventional antibiotics was assessed. A reduction in CV staining > 50% in biofilm biomass by test antimicrobial agents was noticed as an active antibiofilm agent. All HNP-1 analogs displayed a significant reduction of *E*. *coli* biofilm formation not only at the MIC but also at the sub-MICs. However, 2Abz^23^S^29^ and HNP1ΔC18A peptides failed to show > 50% reduction in biofilm biomass of PAO1 at the MICs. The inhibitory activity of the peptide below the MIC values may propose that the anti-biofilm effect of HNP-1 analogs was due to the biofilm-specific mechanisms rather than the direct killing of planktonic bacterial cells. AMPs may inhibit the biofilm formation by direct killing the planktonic bacteria and diminish bacterial adherence by coating the bacteria or by covering the surfaces [24]. When AMPs show the anti-biofilm activity only in concentrations close to their MICs, a direct killing of bacteria is the main effect on biofilm inhibition [25]. It should be noted that HDPs even at concentrations lower than MIC, are capable of killing a significant amount of bacteria, so the specific anti-biofilm activity should be expressed cautiously [26]. We also evaluated the effects of peptides against preformed biofilms because mature biofilms have sticky exopolymeric substances (EPS) that are more challenging to target than the early biofilms [27]. Surprisingly, all test peptides significantly inhibited 24 h-old biofilms of *E*. *coli* up to 50% at sub MIC values. Between the studied peptides, only 2Abz^14^S^29^ at MIC significantly disrupted the biofilm mass, and the other ones, 2Abz^23^S^29^ and HNP1ΔC18A needed higher concentration for disruption of PAO1 mature biofilm. We should point out that CV stained both embedded bacteria and EPS [28]. Therefore, it is suggested that metabolic assay should be used to determine the number of live bacteria of biofilm in future studies. Nowadays, researchers believe that new anti-biofilm therapeutics have to provide opportunities for second immune responses to fight infections by decreasing or delaying biofilm formation, and it is not necessary to eradicate them [29]. The anti-biofilm activity of AMPs at high toxic concentrations and their short life makes them unsuitable as a stand-alone biofilm treatment. So the combination of peptides with antibiotics makes bacterial biofilms easier targets for these agents [30]. Following this approach, we tested the anti-biofilm ability of combined peptides and existing antibiotics. The combination of HNP-1 analogs peptides with amoxicillin or cefixime increased the activity of antibiotics to target *E*. *coli* biofilms, both at the early stages of growth and in their later stages of growth. To confirm the type of antibiofilm efficacy of test peptides in combination with antibiotics, kill kinetic studies need to be investigated in the future, particularly when synergism is observed. According to the species of bacteria, the effects of AMPs in combination with antibiotics might be notably different, so further studies have to be performed with more strains to obtain definitive results.

The results indicated that the test truncated HNP-1 analogs, 2Abz^14^S^29^, 2Abz^23^S^29^, and HNP1ΔC18A may use as a potential source for the treatment of biofilm-associated bacterial diseases, including UTI caused by MDR uropathogenic *E*. *coli*. This effect could be enhancing if the combination of peptide and antibiotic is synergistic, and due to the synergistic effect, the concern over resistance and toxicity will be resolved. Further studies in animal models are necessary for their clinical applications.

## Supporting information

S1 Table Minimum Inhibitory Concentration (MIC) values of test antimicrobial agents against twenty clinical isolates used in this study with their biofilm profiles. (DOCX)

S2 Table Evaluation for synergy of combinations consisting of both antimicrobial peptide and antibiotics against MDR clinical isolates of E. coli (n = 20). AMX: Amoxicillin; CFM: Cefixime; CIP: Ciprofloxacin; NOR: Norfloxacin; MICA, MIC of one peptide alone; MICB, MIC of one antibiotic alone; MICA combination, MIC of one peptide in the most effective combination; MICB combination, MIC of one antibiotic in the most effective combination; ΣFIC ≤ 0.5, synergistic;1≥ΣFIC> 0.5, additive; 1< ΣFIC> 4.0, indifference, ΣFIC> 4.0, antagonistic.
(DOCX)

S1 Fig Biofilm inhibition by individual antimicrobial peptides (2Abz^14^S^29^, 2Abz^23^S^29^, and HNP1ΔC18A) is shown in panels B. Untreated bar indicates *P*. *aeruginosa* PAO1 biofilm without any antimicrobial agent. Data show the means ± SD of three independent experiments. An asterisk indicates the statistically significant difference between antimicrobial agent and untreated control as measured by Student’s t-test (*p < 0.05, **p < 0.01 and ***p < 0.001). Percent numbers indicate % reduction of biofilm.
(TIFF)

S2 Fig Biofilm eradication by individual antimicrobial peptides (2Abz^14^S^29^, 2Abz^23^S^29^, and HNP1ΔC18A) is shown in panel A. Untreated bar indicates *P*. *aeruginosa* PAO1 biofilm without any antimicrobial agent. Data show the means ± SD of three independent experiments. An asterisk indicates the statistically significant difference between antimicrobial agent and untreated control as measured by Student’s t-test (*p < 0.05, **p < 0.01 and ***p < 0.001). Percent numbers indicate % reduction of biofilm.
(TIFF)
