# Supplementary material for: Inhibition and eradication activity of truncated α-defensin analogs against multidrug resistant uropathogenic Escherichia coli biofilm
Source: PLoS One. 2020 Jul 14;15(7):e0235892. doi: 10.1371/journal.pone.0235892 (PMC7360030; doi:10.1371/journal.pone.0235892)
Supplement: S1 Table — (DOCX) [file pone.0235892.s001.docx]

| **Isolate number** | **2Abz^14^S^29^** | **2Abz^23^S^29^** | **HNP-1∆C18A** | **AMX** | **CFM** | **CIP** | **NOR** | **24 h-old biofilm** |
| --- | --- | --- | --- | --- | --- | --- | --- | --- |
| 1 | 62.5 | 125 | 125 | >256 | >256 | 32 | 128 | OD_570_˂1 |
| 2 | 62.5 | 125 | 125 | >256 | >256 | 32 | 128 | OD_570_˂1 |
| 3 | 62.5 | 125 | 125 | >256 | >256 | 32 | 128 | OD_570_˂1 |
| 4 | 62.5 | 125 | 125 | >256 | >256 | 32 | 128 | OD_570_˂1 |
| 5 | 62.5 | 125 | 125 | >256 | >256 | 32 | 128 | OD_570_˂1 |
| 6 | 62.5 | 125 | 125 | >256 | >256 | 32 | 128 | OD_570_˂1 |
| 7 | 62.5 | 125 | 125 | >256 | >256 | 32 | 128 | OD_570_˂1 |
| 8 | 62.5 | 125 | 125 | >256 | >256 | 32 | 128 | OD_570_˂1 |
| 9 | 62.5 | 125 | 125 | >256 | >256 | 32 | 128 | OD_570_˂1 |
| 10 | 62.5 | 125 | 125 | >256 | >256 | 32 | 128 | OD_570_˂1 |
| 11 | 62.5 | 125 | 125 | >256 | >256 | 32 | 128 | OD_570_˂1 |
| 12 | 62.5 | 125 | 125 | >256 | >256 | 32 | 256 | OD_570_˂1 |
| 13 | 62.5 | 125 | 125 | >256 | >256 | 64 | 256 | OD_570_˂1 |
| 14 | 125 | 250 | 250 | >256 | >256 | 64 | 256 | OD_570_˂1 |
| 15 | 125 | 250 | 250 | >256 | >256 | 64 | 256 | OD_570_˂1 |
| 16 | 125 | 250 | 250 | >256 | >256 | 64 | 256 | OD_570_˂1 |
| 17 | 125 | 250 | 250 | >256 | >256 | 64 | 256 | OD_570_˂1 |
| 18 | 125 | 250 | 250 | >256 | >256 | 64 | 256 | OD_570_˂1 |
| 19 | 125 | 250 | 250 | >256 | >256 | 64 | 256 | OD_570_˂1 |
| 20 | 125 | 250 | 250 | >256 | >256 | 64 | 256 | 1.5˂OD_570_˂2 |
