# Supplementary material for: Inhibition and eradication activity of truncated α-defensin analogs against multidrug resistant uropathogenic Escherichia coli biofilm
Source: PLoS One. 2020 Jul 14;15(7):e0235892. doi: 10.1371/journal.pone.0235892 (PMC7360030; doi:10.1371/journal.pone.0235892)
Supplement: S2 Table — AMX: Amoxicillin; CFM: Cefixime; CIP: Ciprofloxacin; NOR: Norfloxacin; MICA, MIC of one peptide alone; MICB, MIC of one antibiotic alone; MICA combination, MIC of one peptide in the most effective combination; MICB combination, MIC of one antibiotic in the most effective combination; ΣFIC ≤ 0.5, synergistic;1≥ΣFIC> 0.5, additive; 1< ΣFIC> 4.0, indifference, ΣFIC> 4.0, antagonistic. (DOCX) [file pone.0235892.s002.docx]

**S2 Table. Evaluation for synergy of combinations consisting of both antimicrobial peptide and antibiotics against MDR clinical isolates of *E. coli* (n=20)*.***

| **Isolate 1-11** |  | **ΣFIC = FIC (A) (MIC of A_in Combination_ /MIC of A _alone_) + FIC (B) (MIC of B_in Combination_/MIC of B _alone_ )** | **Interpretation** |
| --- | --- | --- | --- |
| 2Abz^14^S^29^ |  | 31.25/62.5+32/1024=0.53 | additive |
| 2Abz^23^S^29^ | **AMX or CFM** | 62.5/125+32/1024=0.53 | additive |
| HNP-1∆C18A |  | 62.5/125+32/1024=0.53 | additive |
| 2Abz^14^S^29^ |  | 62.5/62.5+32/128=1.25 | Indifferent |
| 2Abz^23^S^29^ | **NOR** | 125/125+32/128=1.25 | Indifferent |
| HNP-1∆C18A |  | 125/125+32/128=1.25 | Indifferent |
| 2Abz^14^S^29^ |  | 62.5/62.5+8/32=1.25 | Indifferent |
| 2Abz^23^S^29^ | **CIP** | 125/125+8/32=1.25 | Indifferent |
| HNP-1∆C18A |  | 125/125+8/32=1.25 | Indifferent |
| **Isolate 12** |  |  |  |
| 2Abz^14^S^29^ |  | 31.25/62.5+32/1024=0.53 | additive |
| 2Abz^23^S^29^ | **AMX or CFM** | 62.5/125+32/1024=0.53 | additive |
| HNP-1∆C18A |  | 62.5/125+32/1024=0.53 | additive |
| 2Abz^14^S^29^ |  | 62.5/62.5+64/256=1.25 | Indifferent |
| 2Abz^23^S^29^ | **NOR** | 125/125+64/256=1.25 | Indifferent |
| HNP-1∆C18A |  | 125/125+64/256=1.25 | Indifferent |
| 2Abz^14^S^29^ |  | 62.5/62.5+8/32=1.25 | Indifferent |
| 2Abz^23^S^29^ | **CIP** | 125/125+8/32=1.25 | Indifferent |
| HNP-1∆C18A |  | 125/125+8/32=1.25 | Indifferent |
| **Isolate 13** |  |  |  |
| 2Abz^14^S^29^ |  | 31.25/62.5+32/1024=0.53 | additive |
| 2Abz^23^S^29^ | **AMX or CFM** | 62.5/125+32/1024=0.53 | additive |
| HNP-1∆C18A |  | 62.5/125+32/1024=0.53 | additive |
| 2Abz^14^S^29^ |  | 62.5/62.5+64/256=1.25 | Indifferent |
| 2Abz^23^S^29^ | **NOR** | 125/125+64/256=1.25 | Indifferent |
| HNP-1∆C18A |  | 125/125+64/256=1.25 | Indifferent |
| 2Abz^14^S^29^ |  | 62.5/62.5+8/64=1.125 | Indifferent |
| 2Abz^23^S^29^ | **CIP** | 125/125+8/64=1.125 | Indifferent |
| HNP-1∆C18A |  | 125/125+8/64=1.125 | Indifferent |
| **Isolate 14-20** |  |  |  |
| 2Abz^14^S^29^ |  | 62.5/125+32/1024=0.53 | additive |
| 2Abz^23^S^29^ | **AMX or CFM** | 125/250+32/1024=0.53 | additive |
| HNP-1∆C18A |  | 125/250+32/1024=0.53 | additive |
| 2Abz^14^S^29^ |  | 125/125+64/256=1.25 | Indifferent |
| 2Abz^23^S^29^ | **NOR** | 250/250+64/256=1.25 | Indifferent |
| HNP-1∆C18A |  | 250/250+64/256=1.25 | Indifferent |
| 2Abz^14^S^29^ |  | 125/125+8/64=1.125 | Indifferent |
| 2Abz^23^S^29^ | **CIP** | 250/250+8/64=1.125 | Indifferent |
| HNP-1∆C18A |  | 250/250+8/64=1.125 | Indifferent |

AMX: Amoxicillin; CFM: Cefixime; CIP: Ciprofloxacin; NOR: Norfloxacin; MICA, MIC of one peptide alone; MICB, MIC of one antibiotic alone; MICA _combination_, MIC of one peptide in the most effective combination; MICB combination, MIC of one antibiotic in the most effective combination; ΣFIC ≤ 0.5, synergistic;1≥ΣFIC> 0.5, additive; 1< ΣFIC> 4.0, indifference, ΣFIC> 4.0, antagonistic.
